# Serum osteoprotegerin level is positively associated with peripheral artery disease in patients with peritoneal dialysis

**DOI:** 10.1080/0886022X.2020.1714654

**Published:** 2020-01-17

**Authors:** Wei-Chen Lin, Jen-Pi Tsai, Yu-Hsien Lai, Yu-Li Lin, Chiu-Huang Kuo, Chih-Hsien Wang, Bang-Gee Hsu

**Affiliations:** a Division of Nephrology, Hualien Tzu Chi Hospital, Buddhist Tzu Chi Medical Foundation, Hualien, Taiwan; b School of Medicine, Tzu Chi University, Hualien, Taiwan; c Division of Nephrology, Department of Internal Medicine, Dalin Tzu Chi Hospital, Buddhist Tzu Chi Medical Foundation, Chiayi, Taiwan

**Keywords:** Ankle-brachial index, osteoprotegerin, peripheral arterial disease, peritoneal dialysis

## Abstract

Osteoprotegerin (OPG) is a potential biomarker of cardiovascular disease complications and severity. Peripheral arterial disease (PAD) is associated with an increased risk of death in patients on peritoneal dialysis (PD). Therefore, this study aimed to evaluate the relationship between serum OPG levels and PAD by measuring the ankle-brachial index (ABI) of patients on PD. A commercial enzyme-linked immunosorbent assay kit was used to measure OPG values. Left or right ABI values of <0.9 were categorized as the low ABI group. Among 70 patients on PD, 13 (18.6%) were categorized in the low ABI group. Patients in the low ABI group had higher prevalence of diabetes mellitus (*p* = .044) and higher serum C-reactive protein (CRP) (*p* < .001) and OPG levels (*p* < .001) but lower creatinine (*p* = .013) and peritoneal Kt/V (*p* = .048) levels than those in the normal ABI group. Results of multivariable logistic regression analysis revealed that OPG [adjusted odds ratio (aOR) 1.027, 95% confidence interval (CI) 1.010–1.045, *p* = .002] and CRP (aOR 1.102, 95% CI 1.006–1.207, *p* = .037) levels were independent predictors of PAD in patients on PD. OPG can also be used to predict PAD development with the area under the receiver operating characteristic curve of 0.823 (95% CI: 0.714–0.904, *p* < .001) in patients on PD. Therefore, serum OPG and CRP levels can be considered as risk factors for PAD development in patients on PD.

## Introduction

Peripheral artery disease (PAD), which affects approximately 202 million people worldwide, is a condition affecting the atherosclerosis process involving arteries other than the coronary or intracranial arteries [1]. Studies have shown that risk factors such as age, smoking, diabetes mellitus (DM), hypertension (HTN), and dyslipidemia resulted in a rapid increased prevalence of PAD in the last 2 decades [1,2]. Prevalence of PAD in patients who underwent peritoneal dialysis (PD) was higher than that in the general population, and the presence of symptomatic or asymptomatic PAD measured using the ankle-brachial index (ABI) could be a predictor for future loss of residual renal function, cardiovascular (CV) disease, and overall mortality [3,4].

Osteoprotegerin (OPG), which is produced by various organs such as the lungs, intestines, kidneys, bones, heart, and vessels, is a member of the tumor necrosis factor receptor superfamily [5]. Through binding to the receptor activator nuclear factor κ-B ligand, OPG could regulate the bone remodeling balance and even modulate the process of arterial calcification [5,6]. A systematic review showed that serum OPG was associated with the presence and severity of atherosclerosis, including coronary artery disease (CAD), acute coronary syndrome, and CVD [7]. In patients with DM, the serum OPG level could be a predictor for the presence and severity of PAD measured using ABI or color Doppler ultrasonic imaging after adjusting the traditional CV risk factors [8,9]. In patients with chronic kidney disease (CKD), the serum OPG level progressively elevates as the renal function deteriorates, positively correlates with inflammatory markers and intimal-media thickness of carotid arteries, and is most importantly associated with survival [10,11]. In addition, the OPG level was significantly correlated with PAD after renal transplantation [12] and was considered as the strongest predictor of arterial calcification progression in patients on PD [13]. Having PAD was a predictor for further CV morbidity and mortality in the general population, patients with DM, and even those with CKD; however, studies regarding the relationship between the serum OPG level and PAD measured using ABI in patients on PD remained limited. Thus, this study aimed to examine risk factors of PAD in patients on PD and to determine the role of serum OPG levels in these patients.

## Materials and methods

### Patients

A total of 70 patients who underwent PD for at least 3 months at Hualien and Dalin Tzu Chi Hospitals from June 2015 to October 2016 were recruited. Blood pressures (BPs) of all patients were measured by trained staff in the morning using standard mercury sphygmomanometers with appropriate cuff sizes after allowing the patient to sit and rest for at least 10 min. Systolic BP (SBP) and diastolic BP (DBP) values obtained 3 times at 5-min intervals were averaged. This study was approved by the Protection of Human Subjects Institutional Review Board of Tzu Chi University and Hospital and was conducted under the tenets of the Helsinki Declaration. Signed informed consents were obtained from all patients before participating in the study. Patients with acute infection, malignancy, acute myocardial infarction, pulmonary edema, heart failure, or taking cilostazol or pentoxifylline during the blood sampling period or those who refused to sign the informed consent were excluded. Among the included patients, 51 received continuous ambulatory PD (CAPD, Dianeal, Baxter Health Care, Taiwan), with 3–5 dialysate exchanges per day, and the remaining 19 patients received 4–5 dialysate exchanges each night using an automated device (automated PD, APD). Weekly fractional clearance index values for urea (weekly Kt/V), peritoneal Kt/V, total creatinine clearance, and peritoneal creatinine clearance were obtained from medical records.

### Anthropometric analysis

All anthropometric variables were measured in the morning with patients fasting overnight and without dialysate in the abdominal cavity. Body weights and heights were measured by trained staff with patients in light clothing and without shoes and recorded to the nearest 0.5 kg and 0.5 cm, respectively. Body mass index was calculated as weight (kg) divided by height-squared (m^2^).

### Biochemical investigations

Biochemical tests were performed on morning samples obtained after an overnight 8–10 h fasting before the dialysis exchange. Approximately 5 mL of fasting blood samples were obtained and immediately centrifuged at 3000 × *g* for 10 min. Within 1 h of collection, blood samples were stored at 4 °C and used for biochemical analyses. An autoanalyzer (Siemens Advia 1800, Siemens Healthcare GmbH, Henkestr, Germany) was used to measure serum total calcium, phosphorus, fasting glucose, albumin, blood urea nitrogen, creatinine, total cholesterol, triglyceride (TG), and C-reactive protein (CRP) levels. A commercially available enzyme immunoassay or enzyme-linked immunosorbent assays were used to measure serum intact parathyroid hormone (iPTH) (Diagnostic Systems Laboratories, Texas, USA) and OPG (eBioscience Inc., San Diego, CA, USA) levels, respectively [12].

### ABI measurements

ABI values obtained using an oscillometric method were used to measure BP in both arms and ankles (VaSera VS-1000; Fukuda Denshi Co, Ltd, Tokyo, Japan) [12,14]. ABI values are technically the measurement of pressures at the brachialis, dorsalis pedis, posterior tibialis and the ABI itself should be the ratio of higher ankle SBP (dorsalis pedis or posterior tibial artery) in each lower extremity by the higher of the two brachial artery SBP. Occlusion and monitoring cuffs were appropriately used in all 4 limbs of patients in supine position. The real-time electrocardiography was recorded for more than 15 min. Accordingly, PAD was diagnosed based on an ABI of <0.9, and right or left ABI values of <0.9 were defined as low ABI group as in our previous study [12,14,15].

### Statistical analysis

The Kolmogorov–Smirnov test was used to measure data normality. Normally distributed data were expressed as mean ± standard deviation, and two-tailed Student’s independent t-tests were used for between-patient comparisons. Non-normally distributed data were expressed as medians with interquartile ranges and compared using the Mann–Whitney U test (TG, fasting glucose, iPTH, and CRP) between patients. Categorical data were expressed as number with percentage and compared using the χ^2^ test between patients. Variables significantly associated with PAD in patients on PD were tested for independence using the multivariable logistic regression analysis. The receiver operating curve (ROC) was used to calculate the area under the curve (AUC) to identify the most accurate cutoff value of OPG to predict PAD in patients on PD. All statistical analyses were performed using the SPSS software for Windows (version 19.0; SPSS, Chicago, IL, USA). A *p* value of <.05 was considered statistically significant.

## Results

The characteristics of 70 patients on PD are shown in Table 1. Among them, 26 (37.1%) had DM and 44 (62.9%) had HTN. Thirteen patients on PD (18.6%) were categorized into the low ABI group. PAD prevalence was higher in patients on PD with DM than those without (*p* = .044). Serum CRP (*p* < .001) and OPG (*p* < .001) levels were higher but serum creatinine (*p* = .013) and peritoneal Kt/V (*p* = .048) levels were lower in patients on PD in the low ABI group than those in the normal ABI group. No statistically significant difference was observed in gender, coexisting HTN, smoking, or use of CAPD model between the 2 groups.

**Table 1.** Clinical variable of the 70 PD patients in the normal or low ABI group.

Characteristic	All participants	Normal ABI group	Low ABI group	*p* Value
	(*n* = 70)	(*n* = 57)	(*n* = 13)	
Age (years)	56.41 ± 15.30	56.21 ± 15.59	57.31 ± 14.53	.817
Peritoneal dialysis duration (months)	53.76 ± 42.06	54.30 ± 37.07	51.38 ± 61.24	.824
Female, *n* (%)	41 (58.6)	31 (54.4)	10 (76.9)	.137
Diabetes, *n* (%)	26 (37.1)	18 (31.6)	8 (61.5)	.044*
Hypertension, *n* (%)	44 (62.9)	38 (66.7)	6 (46.2)	.167
CAPD model, *n* (%)	51 (72.9)	43 (75.4)	8 (61.5)	.309
Smoking, *n* (%)	9 (12.9)	6 (10.5)	3 (23.1)	.222
ACEi/ARB, *n* (%)	32 (45.7)	29 (50.9)	3 (23.1)	.132
CCB, *n* (%)	30 (42.9)	27 (47.4)	3 (23.1)	.198
Beta blocker, *n* (%)	28 (40)	23 (40.4)	5 (38.5)	.851
statin, *n* (%)	17 (24.3)	13 (22.8)	4 (30.8)	.806
insulin, *n* (%)	11 (42.3)	8 (44.4)	3 (37.5)	.921
OHA, *n* (%)	14 (53.8)	10 (55.6)	4 (50)	.870
Body mass index (kg/m^2^)	24.50 ± 4.27	24.40 ± 3.81	24.94 ± 6.07	.683
Left ankle-brachial index	1.07 ± 0.17	1.13 ± 0.13	0.84 ± 0.13	<.001*
Right ankle-brachial index	1.08 ± 0.15	1.13 ± 0.12	0.87 ± 0.10	<.001*
Systolic blood pressure (mmHg)	144.11 ± 24.49	144.93 ± 22.64	140.54 ± 32.26	.563
Diastolic blood pressure (mmHg)	84.90 ± 12.99	85.33 ± 11.98	83.00 ± 17.17	.563
Albumin (mg/dL)	3.73 ± 0.37	3.74 ± 0.39	3.67 ± 0.28	.543
Total cholesterol (mg/dL)	171.41 ± 38.21	174.07 ± 39.46	159.77 ± 30.80	.226
Triglyceride (mg/dL)	147.00 (100.50–226.50)	150.00 (93.50–222.50)	131.00 (114.00–236.50)	.892
Fasting glucose (mg/dL)	106.00 (95.75–137.25)	104.00 (95.00–127.00)	122.00 (101.50–149.50)	.123
Blood urea nitrogen (mg/dL)	59.56 ± 18.61	60.16 ± 19.62	56.92 ± 13.61	.575
Creatinine (mg/dL)	11.17 ± 3.00	11.59 ± 2.86	9.34 ± 2.98	.013*
Total calcium (mg/dL)	9.15 ± 0.79	9.23 ± 0.79	8.80 ± 0.72	.078
Phosphorus (mg/dL)	5.16 ± 1.35	5.18 ± 1.37	5.12 ± 1.32	.886
Intact parathyroid hormone (pg/mL)	247.16 (91.40–534.35)	213.62 (81.93–507.16)	313.60 (201.70–576.10)	.283
C-reactive protein (mg/dL)	0.27 (0.07–0.96)	0.19 (0.06–0.42)	1.46 (0.81–1.75)	<.001*
Osteoprotegerin (pg/mL)	182.91 ± 77.08	162.36 ± 55.17	273.03 ± 95.66	<.001*
Weekly Kt/V	2.09 ± 0.40	2.11 ± 0.40	1.98 ± 0.36	.276
Peritoneal Kt/V	1.80 ± 0.44	1.85 ± 0.42	1.58 ± 0.46	.048*
Total clearance of creatinine (L/week)	57.32 ± 23.46	57.29 ± 25.33	57.43 ± 13.04	.985
Peritoneal clearance of creatinine (L/week)	42.42 ± 15.87	43.13 ± 15.74	39.30 ± 16.72	.437

Continuous variables are reported as mean ± standard deviation or median and interquartile range and compared with a *t*-test Mann–Whitney *U*-test, as appropriate. Categorical variables are reported as number (%) compared with the chi-square test. ABI: ankle-brachial index; CAPD: continuous ambulatory peritoneal dialysis; Weekly Kt/V: weekly fractional clearance index for urea.

*p < .05 was considered statistically significant.

**Figure 1.**
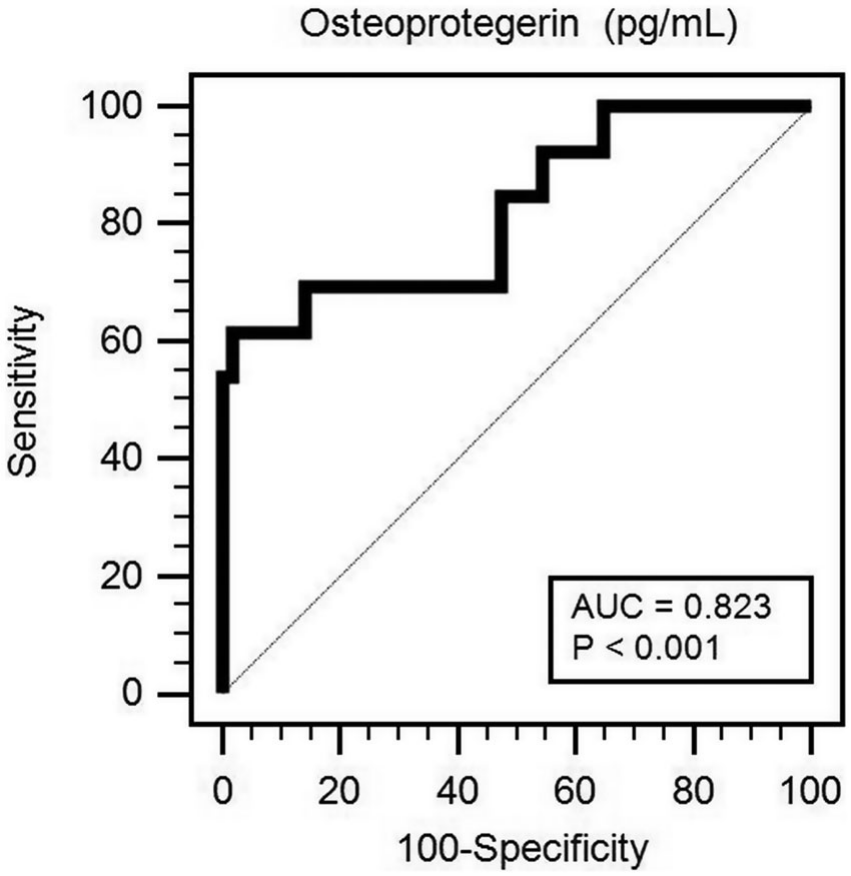
Receiver operating characteristic (ROC) curve analysis to predict PAD of 70 PD patients. The area under the ROC curve (AUC) indicates the diagnostic power of OPG at predicting PAD of PD patients.

After adjusting for confounding factors (DM, log-CRP, peritoneal Kt/V, creatinine, and OPG), the multivariable logistic regression analysis showed that the serum OPG (adjusted odds ratio [a OR] 1.027, 95% confidence interval [CI] 1.010–1.045, *p* = .002) and log-CRP levels (aOR 1.102; 95% CI, 1.006–1.207; *p* = .037) were significantly associated with and identified as independent predictors of PAD in patients on PD (Table 2). Furthermore, the ROC curve plotting for PAD prediction revealed that AUC for OPG was 0.823 (95% CI, 0.714–0.904; *p* < .001) (Figure 1).

**Table 2.** Risk factors for the development of PAD of PD patients.

Variables	Adjusted odds ratio	95% confidence interval	*p* Value
Osteoprotegerin (pg/mL) (each increase of 1 pg/mL)	1.027	1.010–1.045	.002*
Log-CRP	1.102	1.006–1.207	.037*

Analysis was done using the multivariable logistic regression analysis adjusted for DM, CRP, peritoneal Kt/V, creatinine, and OPG. CRP was log-transformed due to skew distribution. CRP C-reactive protein, Peritoneal Kt/V, weekly fractional clearance index for urea.

* *p* < .05 was considered statistically significant.

## Discussion

The results in this study showed that patients on PD with low ABI had significantly higher OPG and CRP levels as well as lower creatinine and peritoneal Kt/V. In addition, serum OPG and CRP levels were identified as significant predictors for PAD development in patients on PD.

Evidences showed that the number of patients diagnosed with PAD had significantly increased in the past 2 decades, with 28.7% and 13.1% global increase in the number of low–middle and high income countries and with expected 40% increase of prevalence in China [1,2]. Risk factors contributed to this marked increasing trend were traditional ones, such as age, smoking, DM, HTN, and dyslipidemia, as well as nontraditional one, decreased renal function [16]. In the National Health and Nutrition Examination Survey, participants with estimated creatine clearance of <60 mL/min/1.72 m^2^ had a 2.5-fold higher risk for developing PAD than those with >60 mL/min/1.72 m^2^, independent of traditional factors [16]. With the advancement of CKD, the PAD prevalence also increased as compared with the normal renal function [17]. Moreover, a nationwide cohort study conducted in Taiwan showed that the overall PAD incidences were 24.2, 12.4, and 20.7 per 1,000 person-years with respectively 4.4-fold, 2.78-fold, and 3.94-fold increased risks in the HD, PD, and all ESRD patients, which emphasized renal function as a risk factor for PAD [18]. Besides old age, DM, vintage of dialysis, low serum creatinine level, and hyperlipidemia as possible risk factors of PAD in patients on PD, studies have shown that hypoalbuminemia and low residual renal Kt/V value were also significant predictors for PAD development [19,20]. The low serum creatinine together with hypoalbuminemia, higher CRP and ferritin found in PAD patients might reflect low muscle mass or frailty of patients and chronic inflammation in patients on dialysis [19,20]. Consistent with these studies, in this study PD patients who had low ABI would have lower peritoneal Kt/V, creatine and more percentage of DM compared to normal ABI group.

OPG, a secretory glycoprotein belonging to the tumor necrosis factor receptor family, produced from the CV system, lung, kidney, and bone, was well known to modulate osteoclastogenesis and vascular calcification [5,6,21]. Studies have shown that OPG was associated with atherosclerotic diseases [6, 21]. Increased medial arterial calcification of the aorta and renal arteries could be prevented by transgenic overexpression of OPG in OPG-deficient mice [6,21]. Moreover, OPG ligand and receptor activator of NF-kappa β could be detected in calcified arterial walls, indicating that OPG played a role on arterial calcification progression [6]. Clinical studies showed that the presence and severity of CAD were associated with OPG and later found that increasing serum OPG level could be a significant predictor for arterial stiffness in patients with CAD [22,23]. Patients diagnosed with atherothrombotic and cardioembolic strokes had significantly higher OPG levels than those who had transient ischemic attack and controls, and OPG levels were correlated with stroke severity [24]. Likewise, diabetic patients who had higher OPG levels had approximately one- to twofold increased risk of PAD occurrence and severity, independent of traditional risk factors [8,9]. Moreover, patients with CKD who had higher OPG levels had more advanced renal disease and more pronounced vascular injury, CAD, DM, or all-cause mortality [10,11]. In patients with PAD, the serum OPG level was inversely correlated with ABI and positively correlated with severity grade of PAD, which could be modulated by inflammation [25]. When considering PAD, serum OPG levels were similar to old age, DM, HTN, hypoalbuminemia, or low creatinine level, which were considered as possible risk factors in patients on PD or renal transplantation [12,13]. Consistent with these studies, we found that OPG remained a significant predictor of PAD development with 1.027-fold increased risk in PD patients.

Conventional PD fluid used glucose as the osmotic agent with the possible results of formation of glucose degradation products to induce peritoneal inflammation and oxidative stress [26,27]. Moreover, using bio-incompatible PD fluids could cause inflammation as well as exit site infection and peritonitis [28]. Together, these factors could explain the higher risk of PAD in PD patients. Evidence had shown that patients on HD had non-significant higher levels of OPG than those on PD but patients on both modalities had higher levels than CKD or control patients and the OPG level was significantly correlated with inflammatory markers, such as interleukin-6, albumin, and CRP [10,11]. Moreover, Avila et al. showed that OPG was associated with more severe arterial calcification in PD patients [13]. Studies had shown that OPG levels could be modulated by inflammatory cytokines, and the presence and severity of PAD were correlated with inflammation [10,11,17,20]. In a multicenter prospective study, high-sensitivity CRP was significantly associated with abnormal ABI levels, revealing that inflammation played a role in the PAD pathogenesis [17]. Moreover, in patients on PD who had PAD, lower albumin and higher CRP levels were found respectively than those without PAD [20]. In this study, CRP together with OPG was found as independent significant predictors for PAD in patients on PD, indicating that OPG modulation by inflammation might be correlated with vascular dysfunction in patients on PD.

In DM patients, insulin was found to regulate the OPG production and vascular calcification in human aortic smooth muscle cells [29], and 24-week treatment of pioglitazone, which was a agonist of peroxisome proliferator-activated receptor gamma, could down-regulate serum levels of OPG as well as CRP [30]. In addition, treating patients with DM or CAD with atorvastatin or simvastatin appeared to lower serum OPG and high-sensitivity CRP as well as reduce arterial stiffness, which indicated beneficial effects of statin on vasculature through anti-inflammation [31,32]. Although studies had shown medications could have modulatory effects on vessels, there were no significant differences of medications between the normal or low ABI groups in this study.

One limitation of this study was that this was a cross-sectional study in a single center with limited number of patients on PD without enough power. The second limitation was the absence of case-matched control group; thus, the mechanism between OPG and ABI in patients on PD could not be concluded in this study. Thirdly, by using ABI lower than 0.9 as having PAD in this study might lead to some misdiagnosis in dialysis patients with vascular calcification (non-compressible arteries) and resulted in false negative diagnosis. Therefore, the definite relationship between serum OPG and PAD in patients on PD should be confirmed by further longitudinal studies before confirming the cause-effect relationship.

## Conclusions

In conclusion, the serum OPG level together with CRP was considered as significant predictors of PAD in patients on PD in this study. These findings indicated that OPG, a bone-modulating protein, might have a role in the PAD development of PD patients, but the mechanism remained to be further elucidated.
